# Platelet Count and Early Outcome in Patients with Spontaneous Cerebellar Hemorrhage: A Retrospective Study

**DOI:** 10.1371/journal.pone.0119109

**Published:** 2015-03-17

**Authors:** Ching-Yueh Lin, Chih-Ya Chang, Chia-Hung Sun, Tsung-Ying Li, Liang-Cheng Chen, Shin-Tsu Chang, Yung-Tsan Wu

**Affiliations:** 1 Department of Physical Medicine and Rehabilitation, Tri-Service General Hospital, National Defense Medical Center, No. 325, Sec. 2, Cheng-Kung Road, Neihu District, Taipei, Taiwan; 2 Department of Physical Medicine and Rehabilitation, School of Medicine, National Defense Medical Center, No.161, Sec. 6, Minquan East Road, Neihu District, Taipei, Taiwan; 3 Department of Rehabilitation, Taichung Veterans General Hospital, No. 1650, Sec. 4, Taiwan Boulevard, Taichung, Taiwan; St Michael’s Hospital, University of Toronto, CANADA

## Abstract

**Introduction:**

The importance of coagulation, hematology, and biochemical variables have been investigated in the stroke population but have not been systemically surveyed in cerebellar hemorrhage (CH) population. The aim of the study was to explore the predictive value of these factors for early outcome in this population.

**Materials and Methods:**

Eighty patients with acute spontaneous CH were retrospectively analyzed. Clinical and laboratory data were collected on admission for analysis. The patients were divided by Glasgow outcome scale (GOS) score at discharge into the good outcome group (GOS score 4 or 5) and the poor outcome group (GOS score 1, 2, or 3). The association between early outcome and clinical or laboratory variables were investigated by binary logistic regression.

**Results:**

There were 46 (57.5%) patients in the poor outcome group and 34 (42.5%) in the good outcome group. The platelet count (PC) was significantly lower in the poor outcome group (187.3 ± 53.0 × 10^9^/l) compared with good outcome group (244.9 ± 63.9 × 10^9^/l) (p < 0.001). Moreover, PC (OR 0.97; p = 0.004) was the strong predictor with poor early outcome.

**Conclusions:**

We firstly show that lower PC is the independent predictor for poor early outcome in patients with spontaneous CH.

## Introduction

Spontaneous intracerebral hemorrhage (ICH) accounts for 10–15% of all the strokes, with a high mortality rate of 19% within the first month. Many studies have striven to identify prognostic predictors for ICH because most survivors have poor functional outcome [1,2].

Cerebellar hemorrhage (CH) is the least common form of ICH (5–13% of all ICH), but arguably the most lethal (mortality rate of 20–75%) because of its unique neurological location near the brainstem [3]. Prognostic factors including larger hematoma volumes or diameter, a Glasgow Coma Scale (GCS) ≤8, and imaging findings that reveal the presence of hydrocephalus, intraventricular hemorrhage (IVH), brainstem compression, or basal cistern obligation, for poor outcome [4–11] or early mortality [4,8,12] have been reported in patients with CH. However, these studies primarily focus on clinical and imaging findings.

The importance of coagulation parameters in stroke has been investigated for decades. For instance, a decrease in the platelet count (PC) and an increase in the mean platelet volume (MPV) was independently associated with ischemic stroke and its poor early outcome [13–16]. PC had a significant negative correlation with the infarct size [16]. However, reports concerning the role of coagulation parameters in hemorrhagic stroke are rare. While some studies show that decreasing PC is a risk factor for ICH [14–16] and that PC, in combination with the international normalized ratio (INR), are good predictors for an early outcome [17], others show opposite results [18,19].

Regarding the hematologic and biochemical parameters in acute stroke, hyperglycemia (blood sugar [BS] >150 mg/dl) [20], higher serum uric acid level [21,22], C-reactive protein (CRP) level [23], white blood cell (WBC) count [17], and lower hemoglobin (Hgb) level [24] were found to be predictors for bad outcome or death of ICH. However, the number of CH patients in these studies was small. Hyperglycemia (BS ≥ 140 mg/dl) was recently proved an independent predictor of poor outcome of CH [25].

The effects of coagulation, and hematologic and biochemical variables have not been systemically investigated in a CH population. The aim of the study was to explore the predictive value of these major variables for early outcome in patients with spontaneous CH.

## Materials and Methods

This retrospective study was approved by the Institutional Review Board (IRB)/Ethics Committee of Tri-Service General Hospital, National Defense Medical Center. Data from 80 consecutive patients (August 2004 to May 2012) with spontaneous CH admitted to the Tri-Service General Hospital, Taiwan within 48 h following onset were collected, reviewed, de-identified by the authors, and analyzed anonymously. The authors had access to patients’ records prior to data anonymization, and the IRB/Ethics Committee waived the need for informed consent. None of these 80 patients had a previous disability from any known cause nor did they have a stroke history, other simultaneous extra-CH, or hemorrhagic transformation following an ischemic stroke. All clinical parameters were reviewed, including age, sex, smoking and alcohol consumption, and previous systemic diseases as detailed in the medical charts. In each case, the initial GCS score, BS, systolic blood pressure, diastolic blood pressure, heart rate (HR), and body temperature were measured on admission. Hematologic and biochemical studies to determine WBC count, Hgb level, PC, liver function (aspartate aminotransferase [AST] level), renal function (blood urea nitrogen and creatinine levels), coagulation parameters (prothrombin time [INR] and partial thromboplastin time), and serum sodium (Na) and potassium (K) levels were performed after venous blood withdrawal just prior to computer tomography (CT). Characteristic imaging findings included hemorrhage location, ICH volume (ABC/2 method, where A is the greatest diameter of hemorrhage by CT, B is the largest diameter perpendicular to A, and C is the number of CT slices of the hemorrhage multiplied by the slice thickness) [26], IVH, hydrocephalus, and radiographic signs of brainstem compression (obliterated basal cisterns). Outcomes of these patients at discharge were determined by the Glasgow outcome scale (GOS) [27] and the ICH score was used for analysis (Table 1), as previously described [28].

**Table 1:** Evaluation of five components in ICH score [28].

ICH score component		Points
GCS score		
	3–4	2
	5–12	1
	13–15	0
Age≧80 (years)		
	Yes	1
	No	0
Infratentorial origin of ICH		
	Yes	1
	No	0
ICH volume≧30 (cm^3^)		
	Yes	1
	No	0
IVH		
	Yes	1
	No	0
Total ICH score		0–6

Note:

1. GCS = Glasgow Coma Scale; ICH = intracerebral hemorrhage; IVH = intraventricular hemorrhage.

2. GCS score, initial GCS score on arrival (or after resuscitation); ICH volume, calculated by ABC/2 method; IVH, the presence of IVH on CT imaging.

To determine the predictors of outcome at discharge, we divided the patients into two groups depending on functional outcome: (1) the good outcome group, independently performing activities of daily living (GOS score of 4 or 5) and (2) the poor outcome group, dependent for activities of daily living or death (GOS score of 1, 2, or 3).

All statistical analyses were performed utilizing SPSS 13.0 for Windows. The demographic data and baseline characteristics between good and poor outcome groups were analyzed via Mann—Whitney *U*-test for numerical variables, and the chi-square test and Fisher’s exact test for categorical variables. Potential prognostic factors including clinical parameters, ICH score, and laboratory data were analyzed by the *t*-test. The relationship between PC and hematoma volume was analyzed by testing the Pearson correlation coefficient. Moreover, significant variables (p < 0.05) were entered into a binary logistic regression with forward stepwise regression to determine the prognostic factors for poor outcome at discharge. The independent predictors in the final model were presented as odds ratios (ORs), including 95% confidence intervals (CI). Statistical significance was set at p < 0.05.

## Results

The baseline characteristics and demographic data of 80 patients are shown in Table 2. A total of 17 (21.2%) patients died prior to discharge, with 14 (14/17, 82.3%) of the deaths occurring within the first week following stroke. Of the 63 (78.8%) surviving patients at discharge, we classified 29 (46%) and 34 (54%) into the poor and good outcome groups, respectively. In total, there were 46 (57.5%) patients in the poor outcome group and 34 (42.5%) in the good outcome group.

**Table 2:** The baseline characteristics and comparison of demographic data of the study population.

Variable		Good (GOS 4–5) (n = 34)	Poor (GOS 1–3) (n = 46)	P-value	
Age (year) (SD)		62.0 ± 16.1	68.65 ± 15.31	0.064	
Male/female, n		21.0/13.0	29.0/17.0	0.907	
Smoking, n (%)		15.0 (44.1)	21.0 (45.6)	0.892	
Drinking, n (%)		10.0 (29.4)	19.0 (41.3)	0.317	
Hypertension, n (%)		22.0 (64.7)	35.0 (76.0)	0.266	
Diabetes mellitus, n (%)		8.0 (23.5)	13.0 (28.2)	0.634	
Coronary artery disease, n (%)		10.0 (29.4)	14.0 (30.4)	0.921	
Dyslipidemia, n (%)		1.0 (2.9)	1.0 (2.1)	0.672^**a**^	
Coagulopathy, n (%)		1.0 (2.9)	7.0 (15.2)	0.070	
Lesion site				0.947^**b**^	
	Right, n (%)	15.0 (44.1)	19.0 (41.3)		
	Left, n (%)	15.0 (44.1)	22.0 (47.8)		
	Vermis, n (%)	4.0 (11.8)	5.0 (10.9)		
Hydrocephalus, n (%)		13.0 (38.2)	39.0 (84.7)	0.000	***
Brain stem compression, n (%)		0 (0)	22.0 (47.8)	0.000	***
IVH, n (%)		14.0 (41.1)	33.0 (71.7)	0.006	**
ICH volume≥30 (cm^3^)		4.0 (11.7)	39.0 (84.7)	0.000	***
GCS score				0.000^**b**^	***
	3–4	0 (0)	13.0 (28.3)		
	5–12	6.0 (17.6)	19.0 (41.3)		
	13–15	28.0 (82.4)	14.0 (30.4)		

Note:

1. GOS = Glasgow Outcome Scale; GCS = Glasgow Coma Scale; IVH = intraventricular hemorrhage; SD = standard deviation.

2. ^a^Fisher’s Exact Test was used; ^b^Phi&Cramer’s V was used.

3. Numerical variables were analyzed by Mann—Whitney U-test and categorical variables were analyzed by chi-squared test and Fisher’s exact test.

***P≦0.001;

**P≦0.01.

The mean ± standard deviation (SD) age of the patients was 68.65 ± 15.31 years in the poor outcome group and 62.0 ± 16.1 years in the good outcome group. In addition, we found that the prevalence of co-morbidities such as hypertension (n = 35 [76.0%] vs. n = 22 [64.7%]), diabetes (n = 13 [28.2%] vs. n = 8 [23.5%]), coronary artery diseases (n = 14 [30.4%] vs. n = 10 [29.4%]), and coagulopathy (n = 7 [15.2%] vs. n = 1 [2.9%]) was higher in the poor outcome group, except dyslipidemia (n = 1 [2.1%] vs. n = 1 [2.9%]). None of the above characteristics was significantly different between the good and poor outcome groups. However, results revealed a significant downward trend in the GCS score (GCS score ≤12: n = 32 [69.6%] vs. n = 6 [17.6%]) in the poor outcome group (p < 0.001). Moreover, the prevalence of hydrocephalus (n = 39 [84.7%] vs. n = 13 [38.2%]; p < 0.001), brain stem compression (n = 22 [47.8%] vs. n = 0 [0%]; p < 0.001), IVH (n = 33 [71.7%] vs. n = 14 [41.1%]; p = 0.006), and ICH volume >30 cm^3^ (n = 39 [84.7%] vs. n = 4 [11.7%]; p < 0.001) were also found to be significantly higher in the poor outcome group. Although a negative correlation was found between PC and hematoma volume, it was not statistically significant (r = -0.220; p = 0.06).

The results of the stepwise logistic regression analysis for outcome predictors at discharge are shown in Table 3. Our results indicate that the significant predictive factors for outcome in the poor vs. good outcome groups are as follows: initial HR (94.9 ± 18.2 vs. 79.1 ± 15.9 beats/min, p < 0.001), ICH score (3.7 ± 1.4 vs. 1.6 ± 0.7, p < 0.001), BS (10.47 ± 3.29 (188.6 ± 59.3) vs. 7.44 ± 1.68 (134.1 ± 30.3) mmol/l [mg/dl], p < 0.001), PC (187.3 ± 53.0 vs. 244.9 ± 63.9 × 10^9^/l, p < 0.001), and AST level (34.0 ± 17.4 vs. 26.3 ± 9.1 U/l, p = 0.013). However, only four of the five risk factors were found to significantly correlate with poor outcome at discharge in the stepwise logistic regression analysis: initial HR (OR 1.08; 95% CI 1.02–1.14; p = 0.011), ICH score (OR 3.43; 95% CI 1.54–7.66; p = 0.003), BS (OR 1.04; 95% CI 1.01–1.07; p = 0.011), and PC (OR 0.97; 95% CI 0.95–0.99; p = 0.004). Therefore, based on our results, these four factors should be considered significant predictors of poor outcome in CH.

**Table 3:** The stepwise logistic regression analysis of the study population and the potential factors affecting early outcome at discharge.

Variable	Good (GOS 4–5) (n = 34)	Poor (GOS 1–3) (n = 46)	P-value		Stepwise logistic regression			
					Odds ratio	95% CI	P-value	
Initial SBP (mm Hg) (SD)	183.9 ± 32.4	196.0 ± 43.8	0.159					
Initial DBP (mm Hg) (SD)	101.9 ± 17.3	106.3 ± 29.9	0.403					
Initial HR (beat/min) (SD)	79.1 ± 15.9	94.9 ± 18.2	0.000	***	1.08	(1.02–1.14)	0.011	*
Initial BT (℃) (SD)	36.4 ± 0.8	36.2 ± 0.8	0.293					
ICH score	1.6 ± 0.7	3.7 ± 1.4	0.000	***	3.43	(1.54–7.66)	0.003	**
Blood sugar (mmol/l (mg/dl))	7.4 ± 1.6 (134.1 ± 30.3)	10.4 ± 3.2 (188.6 ± 59.3)	0.000	***	1.04	(1.01–1.07)	0.011	*
White blood cell count (x10^9^/l)	10.3 ± 2.7	11.5 ± 4.3	0.148					
Hemoglobin (mmol/l (g/dl))	8.7 ± 1.4 (14.1 ± 2.3)	8.6 ± 1.3 (13.9 ± 2.1)	0.710					
Platelet count (× 10^9^/l)	244.9 ± 63.9	187.3 ± 53.0	0.000	***	0.97	(0.95–0.99)	0.004	**
BUN level (mmol/l (mg/dl))	6.3 ± 1.6 (17.8 ± 4.5)	6.7 ± 3.8 (19.0 ± 10.9)	0.483					
Creatinine level (μmol/l (mg/dl))	83.8 ± 76.2 (1.1 ± 1.0)	83.8 ± 61.0 (1.1 ± 0.8)	0.917					
AST (U/l)	26.3 ± 9.1	34.0 ± 17.4	0.013	*				
Sodium level (Na) (mmol/l)	138.4 ± 3.4	138.9 ± 3.7	0.575					
Potassium level (K) (mmol/l)	3.6 ± 0.4	3.5 ± 0.5	0.644					
Prothrombin time (s)	11.8 ± 1.8	12.7 ± 4.9	0.248					
Partial thromboplastin time (s)	26.4 ± 3.0	30.9 ± 23.2	0.267					
Prothrombin time (INR)	1.1 ± 0.3	1.3 ± 1.3	0.217					

Note:

1. SBP = systolic blood pressure; DBP = diastolic blood pressure; HR = heart rate; BT = body temperature; ICH = intracranial hemorrhage; BUN = blood urea nitrogen; AST = aspartate aminotransferase.

2. t-test was used to compare the difference between two groups and significant variables was evaluated by binary logistic regression with forward stepwise. The independent risk factors in the final model were presented as odds ratios, including 95% confidence intervals (CI).

***P≦0.001;

**P≦0.01;

* p≦0.05.

## Discussion

The presented study was the first to analyze the predictive value of coagulation parameters, and hematologic and biochemical factors for poor outcome in spontaneous CH. The results revealed that increased HR, hyperglycemia, increased ICH score, and decreased PC at admission are prognostic indicators of poor outcome at discharge in spontaneous CH. This is also the first to report that lower PC and an elevated ICH score can be used as predictors of outcome in CH.

Recently, the effect of PC, MPV, and other coagulation parameters in stroke has been increasingly investigated. PC and MPV have been found to relate to the bleeding time and account for an increased probability of hemostasis and thrombosis if increased beyond normal [29]. Moreover, thrombocytopenia, coagulation disorder, and vascular dysfunction were reported to be risk factors for ICH [30]. Another study demonstrated that a decrease in PC was a predictor for poor outcome in the hemorrhagic stroke [14]. In another study from Taiwan, a lower PC on admission could predict mortality following ICH [17]. Interestingly, the markers of coagulopathy (prolonged INR > 1.4, PTT > 35, or thrombocytopenia) are suggested to be prognostic of neurological deterioration and 30-day mortality in ICH [31]. Despite a number of reports illustrate an opposite opinion on this matter. For example, higher PC was found to predict elevated perihematoma edema, accounting for a poor discharge outcome [19]. Another study suggests that PC did not predict mortality in spontaneous ICH [18]. Regardless of these previous ICH studies, this is the first to demonstrate that decreased PC is an independent predictor for poor early outcome in CH.

It is not uncommon for patients with ICH to have pre-existing or secondary platelet dysfunction, which can lead to disturbances in hemostatic response in addition to rapid hematoma expansion. Decreased platelet aggregation [32] and hypofunction [33] have been reported in patients with hemorrhagic stroke compared with that in non-stroke control subjects. Similarly, in another study, platelet dysfunction associated with anti-platelet agents was found to be a risk factor for CH among all ICH events [34]. Despite these findings, there has been no significant correlation between PC and hematoma size made thus far. It is still unclear whether causal or consequential platelet dysfunction can contribute to the worsening of a patient’s condition in hemorrhagic stroke. Mayda-Domac et al. [14] found no significant correlation between PC and hematoma volume. Some authors have also shown that PC does not correlate with hematoma growth [35,36]. In addition, Kawano-Castillo et al. [37] demonstrated that conventional coagulation assays (PC, PTT, and INR) failed to predict the phenomenon of coagulopathy-associated hematoma enlargement. Although low-grade disseminated intravascular coagulopathy concomitant with PC reduction may occur in ICH when hemorrhage is in the intraventricular or subarachnoid space, the occurrence of these scenarios was still rare [31]. In line with previous studies, a non-significant negative correlation between PC and hematoma size was found in our CH group. The clotting dynamics and coagulation profile surrounding the CH event are topics that should be addressed and explored in future research.

Our results also found that ICH score is a strong predictor of poor outcome in CH. These results are expected, considering that infratentorial origin a component for determining the ICH score. In addition, the other subscales of ICH score (including GCS scale, ICH volume, IVH, and old age) were found to be predictors or risk factors for early poor outcome or mortality in patients with CH [4–12,25]. The current study confirmed these results.

In our finding, an increased HR significantly correlated with poor outcome. However, evidence on the relationship between HR and outcome in the hemorrhagic stroke is scarce and controversial [4,25,38]. For example, HR is prognostic of outcome of hemorrhagic stroke and traumatic brain injury [38]. A higher HR was significantly associated with poor outcome at discharge in patients with CH [25]. However, another published the opposite results [4]. Further research is needed.

None of the hematologic and biochemical variables were found to be significantly associated with a poor outcome except for AST level; however, following logistic regression analysis, AST level was no longer statistically significant. As compared with previous studies investing ICH populations [17,24], we did not find that WBC count and Hgb level were significantly correlated with clinical outcome. Finally, an increased BS level is a prognostic indicator of poor outcome in the current study confirms data from our previous report [25].

Our study did have a few limitations. First, the study sample size is relatively small despite its 8-year duration; this is because CH is relatively rare. Second, patients were enrolled from only one medical center in Taiwan; in addition, there was discrepancy in the duration of hospital stay, which might have introduced some bias. Third, the lack of follow-up after patients were discharged limited our understanding of any potential long-term outcomes. Fourth, the previous standards of medications and surgical interventions were not analyzed because no significant difference was observed between the two groups. Finally, the levels of MPV, uric acid, and CRP were not considered in the analysis, as they are not routinely examined in our facility.

## Conclusions

The present study is the first to show that decreases in PC and increases in the ICH score are independent predictors for poor outcome at the time of discharge in patients with spontaneous CH.
